# Prognosis of local invasive relapses after carcinoma in situ of the breast: a retrospective study from a population-based registry

**DOI:** 10.1007/s10549-022-06807-w

**Published:** 2022-11-23

**Authors:** Samia Kada Mohammed, Tienhan Sandrine Dabakuyo Yonli, Isabelle Desmoulins, Ariane Manguem Kamga, Clémentine Jankowski, Marie-Martine Padeano, Catherine Loustalot, Hélène Costaz, Sylvain Causeret, Karine Peignaux, Magali Rouffiac, Charles Coutant, Laurent Arnould, Sylvain Ladoire

**Affiliations:** 1 Department of Gynaecology and Obstetrics, Assistance Publique des Hôpitaux de Paris (APHP), Jean Verdier Hospital, Avenue du 14 Juillet, 93140 Bondy, France; 2 Breast and Gynaecologic Cancer Registry of Côte d’Or, Epidemiology and Quality of Life Research Unit, Georges-François Leclerc Comprehensive Cancer Centre-UNICANCER, 1 rue du Professeur Marion, 21000 Dijon, France; 3 INSERM U1231, 21000 Dijon, France; 4 Department of Medical Oncology, Georges-François Leclerc Centre, 1 rue du Professeur Marion, 21000 Dijon, France; 5 Department of Surgery, Georges-François Leclerc Centre, 1 rue du Professeur Marion, 21000 Dijon, France; 6 Department of Radiotherapy, Georges-François Leclerc Centre, 1 rue du Professeur Marion, 21000 Dijon, France; 7 University of Burgundy-Franche Comté, 21000 Dijon, France; 8 Unit of Pathology, Department of Tumour Biology and Pathology, Georges-François Leclerc Centre, 1 rue du Professeur Marion, 21000 Dijon, France

**Keywords:** Breast cancer, Local invasive recurrence, DCIS, Ductal carcinoma in situ, Survival, Systemic adjuvant therapies

## Abstract

**Purpose:**

The prognosis of local invasive recurrence (LIR) after prior carcinoma in situ (CIS) of the breast has not been widely studied and existing data are conflicting, especially considering the specific prognosis of this entity, compared to de novo invasive breast cancer (de novo IBC) and with LIR after primary IBC.

**Methods:**

We designed a retrospective study using data from the specialized Côte d’Or Breast and Gynecological cancer registry, between 1998 and 2015, to compare outcomes between 3 matched groups of patients with localized IBC: patients with LIR following CIS (CIS-LIR), patients with de novo IBC (de novo IBC), and patients with LIR following a first IBC (IBC-LIR). Distant relapse-free (D-RFS), overall survival (OS), clinical, and treatment features between the 3 groups were studied.

**Results:**

Among 8186 women initially diagnosed with IBC during our study period, we retrieved and matched 49 CIS-LIR to 49 IBC, and 46 IBC-LIR patients. At diagnosis, IBC/LIR in the 3 groups were mainly stage I, grade II, estrogen receptor-positive, and HER2 negative. Metastatic diseases at diagnosis were higher in CIS-LIR group. A majority of patients received adjuvant systemic treatment, with no statistically significant differences between the 3 groups. There was no significant difference between the 3 groups in terms of OS or D-RFS.

**Conclusion:**

LIR after CIS does not appear to impact per se on survival of IBC.

## Introduction

Ductal carcinoma in situ of the breast (DCIS) accounts for 15% of breast cancers in western countries [1]. Despite carrying good prognosis, with an overall 10-year survival rate of more than 95% with current practices (mastectomy, or breast-conserving surgery followed by radiation), the rate of invasive recurrence can be as high as 15% at 10 years, notably depending on the quality of initial treatment [2]. Logically, patients with local invasive recurrence (LIR) after a prior DCIS have significantly worse breast cancer-specific survival (BCSS) and overall survival (OS) compared to those who do not experience recurrence, with 17-fold higher specific breast cancer mortality, and a 5-fold higher risk of death [3].

Local recurrence after DCIS is invasive in more than half of patients who were initially treated by conservative surgery [4, 5]. Fifteen to 22% of these LIR [6, 7] progress to the lymph nodes and/or to distant metastasis, which can be potentially life-threatening. Although outcomes in patients with non-invasive recurrence are excellent, data are sparse regarding outcomes after invasive recurrence [8], and existing data in the literature are conflicting concerning the prognosis of this entity per se. Indeed, some studies have reported that patients with invasive recurrence after primary DCIS have a particularly poor prognosis [8], while others have reported no difference in prognosis for women who experience invasive breast cancer (IBC), with or without prior DCIS [9]. Therefore, the question of invasive ipsilateral relapse management after DCIS remains unresolved, especially regarding the specific need for additional adjuvant systemic treatment. There is a need to prevent progression to metastasis and death, while at the same time, avoiding overtreatment of these patients. In the current context, where overtreatment is a major issue, there is a compelling need to better describe the prognosis of LIR after DCIS, and to investigate if and how these relapses differ per se from other IBC presentations in terms of long-term outcome.

We performed a retrospective study using data from the specialized Côte d’Or Breast and Gynecological cancer registry (Cote d’Or, France), to compare the outcomes between 3 groups of matched patients with localized invasive breast cancer, namely:

(i) Patients with LIR, presenting as IBC occurring in the ipsilateral breast after local treatment of CIS (CIS-LIR group),
(ii) Patients with de novo IBC, without any history of ipsilateral cancer (de novo IBC group).
(iii) Patients with LIR, presenting as IBC in the ipsilateral breast after treatment of a previous IBC (IBC-LIR group).

The primary objective was to determine the impact of a prior diagnosis of ipsilateral CIS on long-term prognosis of IBC, notably distant relapse-free survival (D-RFS) and OS, by comparing these three groups. The secondary objective was to compare distant relapse-free interval (D-RFI) between groups.

## Materials and methods

### Study population

We performed a retrospective study using data from the specialized French breast and gynecological cancers registry of Côte d’Or, the only registry in France specifically focused on breast and gynecological cancers. This registry has been collecting comprehensive population-based data at the time of diagnosis for all cases of breast and gynecological cancer occurring in residents of the Côte d’Or Department of Eastern France since 1982.

From January 1, 1998, to December 31, 2015, a total of 8186 patients were recorded in the registry with a diagnosis of breast cancer. We excluded patients with other cancer locations; diagnosis before January 1, 1998, and after December 31, 2015; patients aged less than 18 years at diagnosis; patients who had metastatic breast cancer at the time of diagnosis (but we evaluated their proportion in our different study groups); patients with lobular intraepithelial neoplasia 1 (LIN 1) or 2 (LIN2); patients with sarcoma or lymphoma of the breast; and patients who did not meet the definition of any of the 3 study groups.

We finally retained a total of 49 CIS-LIR patients, who were matched with 49 de novo IBC patients, and with 46 IBC-LIR patients.

### Ethics

The Côte d’Or breast and gynecological cancer registry has a permanent authorization from the French national data protection authority (Commission nationale informatique et libertés CNIL N1539818) for data recording. The study was also approved by the French national data protection authority (Commission nationale de l’informatique et des libertés MR003 N_1989764 v. 0).

### Clinicopathological assessment

For each patient, the following data were extracted from the registry database: Age at diagnosis of CIS and IBC, age at recurrence, size of primary tumor for CIS-LIR patients, histology of the primary and second tumors, margin status, pathological stage according to the American Joint Committee on Cancer tumor, node, metastasis (AJCC/TNM) classification system, 8^th^ edition, 2018 [10], tumor grade according to the Scarff-Bloom-Richardson (SBR) classification, estrogen receptor (ER) status, human epidermal growth factor receptor-2 (HER2) status, neoadjuvant chemotherapy, type of surgical treatment (breast-conserving surgery or mastectomy), and adjuvant treatments (chemotherapy, radiotherapy, or endocrine therapy).

Histological tumor grade was based on the criteria of Elston and Ellis [11]. Estrogen receptor (ER) positivity was defined as at least 10% nuclear staining by immunohistochemistry (IHC). HER2 expression was assessed by IHC or fluorescence in situ hybridization (FISH) if necessary. HER2 was considered positive if the IHC score was 3 + and negative if the score was 0 or 1 + based on the staining intensity. If the score was 2 + , further assay with FISH was performed. In each nucleus, the number of *HER2* signals and chromosome 17 centromere signals (chromosome enumeration probe 17 [CEP 17]) were counted. The HER2/CEP 17 ratio was calculated, and a ratio of ≥ 2 was considered to be amplified. If the average of *HER2* signals was greater than 6, the case was considered as amplified, regardless of the HER2/CEP 17 ratio. These cut-offs were in accordance with the American Society of Clinical Oncology/ College of American Pathologists (ASCO/CAP) recommendations [12].

### Constitution of the 3 groups of patients

We allocated patients to one of three groups. The first group comprised patients with local, non-metastatic LIR after prior treatment of CIS in the same breast. The second group consisted of patients treated for a first IBC occurring de novo, without prior ipsilateral CIS. The third group consisted of patients with LIR occurring after prior treatment of a first IBC in the same breast.

CIS-LIR and de novo IBC Groups were matched for IBC characteristics (i.e., characteristics of LIR in CIS-LIR group and of de novo IBC in the second group). The matching criteria were as follows: tumor size T (T0-T2 versus T3-T4), nodal status (N- versus N +), SBR grade (1–2 versus 3), ER status (positive or negative), and HER 2 status (positive or negative).

CIS-LIR and IBC-LIR groups were also matched for IBC characteristics (i.e., characteristics of LIR in both groups). The matching criteria were the same, except for tumor grade, because in most cases, the tumor grade of IBC occurring after local treatment of a first IBC cannot be evaluated by pathologist.

### Endpoint definitions

The primary endpoint was D-RFS [13]. Distant relapse-free survival was defined as the time between the date of diagnosis of the invasive tumor (LIR for CIS-LIR and IBC-LIR groups, or the de novo IBC) and the date of distant metastasis or death, whichever occurred first. Patients alive without distant metastatic relapse were censored as of November 1, 2018, the cut-off date for survival analysis. Secondary endpoints were OS and D-RFI. Overall survival was defined as the time from the date of IBC diagnosis (namely the LIR in CIS-LIR and IBC-LIR groups) and the date of death or last follow-up. For D-RFI, the event studied was distant metastasis in each group. D-RFI was defined as the time from the date of the diagnosis of the IBC (namely the LIR in CIS-LIR and IBC-LIR groups) and the date of distant metastasis.

### Statistical analysis

Clinical data, tumor characteristics, and treatments across the three groups are described as mean ± standard deviation (SD) or median [range] for quantitative variables, and number (percentage) for qualitative variables. Groups were compared using the Chi2 for paired series (or Fisher’s exact) test for qualitative variables, and the Kruskal Wallis test or analysis of variance (ANOVA) for quantitative variables, as appropriate. The cut-off date for survival analyses was set at November 1^st^, 2018. Median follow-up was calculated using the reverse Kaplan Meier method [14]. Survival curves were constructed with the Kaplan Meier method, for overall survival and distant relapse-free survival. The log rank test was used to compare survival curves. Finally, multivariable regression analysis using Cox’s proportional hazards model was used to calculate hazard ratios with 95% confidence interval (95% CI) for OS and D-RFS. Multivariable analysis included all matching variables, plus age and histologic type of the tumor at relapse.

All statistical analyses were performed using STATA^©^ statistical software version 13.1 (StataCorp, College Station, Texas, USA). A *p*-value < 0.05 was considered statistically significant.

## Results

### Patients and relapses

Over the period 1998 to 2015, 8186 women living in Côte d’Or were diagnosed with breast cancer and enrolled in the registry database of breast and other gynecological cancers of Côte d’Or. Fifty-five (0.67% of the whole cohort) had CIS followed by an invasive ipsilateral recurrence, of whom 6 (10.9% of all patients with ILR after CIS) had metastatic disease at the diagnosis of ILR. Six thousand five hundred and six patients (79.5% of the whole cohort) had primary IBC without any personal history of mammary carcinoma, including 394 (6%) patients with metastatic disease at the time of the first IBC. Two hundred and fourteen women (2.6% of the whole cohort) had an LIR after a primary IBC including 2 (0.9% of the patients with LIR after primary IBC) with metastatic disease at the time of the second IBC. There were statistically significantly more patients who relapsed with metastatic disease (*p* = 0.002) in CIS-LIR and IBC-LIR groups. All patients with metastatic breast cancer at diagnosis of IBC were excluded from the matching procedure for the present study.

A total of 49 patients with LIR after primary CIS (CIS-LIR) were matched with patients with de novo IBC, and with 46 patients with ILR after previous IBC (IBC-LIR). The mean and median time from diagnosis of CIS to the occurrence of ipsilateral ILR in Group 1 were 6.4 ± 3.8 years (3.77) and 6.9 years [1.01; 16.09], respectively.

The mean and median time from initial IBC diagnosis to ILR after primary IBC for Group 3 were 5.7 ± 3.7 years and 5.3 years [1.42; 15.22], respectively. There was no statistically significant difference in time to LIR between CIS-LIR and IBC-LIR groups (*p* = 0.3194).

### Clinical and tumor characteristics of the patients and primary CIS in CIS-LIR group

The clinical and tumor characteristics of the 49 patients in CIS-LIR, and of their primary CIS, are detailed in Table 1. The mean age was 53.4 years and the average tumor size was 18 ± 17 mm. Forty-five (91.8%) tumors were DCIS and 4 tumors (8.2%) were lobular carcinoma in situ (LCIS). Forty-three (87.8%) tumors had negative margins, 3 (6.1%) had positive margins, 2 (4.1%) had close margins < 1 mm, and one tumor had unknown margins. Concerning patients with positive, close, or unknown margins, none had re-excision, 3 patients were treated by radiotherapy, and 3 patients were treated without radiotherapy.

**Table 1:** Clinical and tumor (CIS) characteristics of CIS-LIR group (*N* = 49)

Characteristics	Patients (N = 49)	%
Age (years)		
Mean (SD)	53.4 (11.9)	
Median [Min; Max]	52 [35; 83]	
Missing data	3	
Tumor size (mm)		
Mean (SD)	18 (17)	
Median [Min; Max]	14 [3; 65]	
Missing data	17	
Histology		
DCIS	45	91.8
LCIS	4	8.2
Margins status		
Negative	43	87.8
Positive	3	6.1
Close < 1 mm	2	4.1
Missing data	1	2
Treatment		
Surgery		
Yes	49	100
Lumpectomy	38	77.6
Mastectomy	11	22.4
No	0	0
Adjuvant radiotherapy		
Yes	26	53.1
No	23	46.9
Adjuvant endocrine therapy		
Yes	2	4.1
No	47	95.9

The treatment for the primary CIS in these patients is summarized in Table 1. All were treated by surgery, with 38 patients (77.6%) undergoing lumpectomy, and 11 patients (22.4%) undergoing mastectomy. Twenty-six patients (53.1%) received radiotherapy after surgery and 23 patients (46.9%) did not. Two patients (4.1%) received adjuvant endocrine therapy, whereas 47 patients (95.9%) did not.

### Comparison of clinical and pathological characteristics of IBC between CIS-LIR, de novo IBC, and IBC-LIR groups

The clinical and pathological characteristics of the patients and the IBCs are summarized in Table 2. These are characteristics of ILR for CIS-LIR patients, of the de novo IBC for the second group, and of the LIR in IBC-LIR group. At the definitive pathological examination, IBC was mainly of stage I: pT0-T1, N0, grade II with positive ER and HER2-negative status.

**Table 2:** Clinical and tumor characteristics of IBC in all groups

Characteristics	CIS-IBC group (*N* = 49)	de novo IBC group (*N* = 49)	IBC-LIR group (*N* = 46)	*p*-value
Age (years)	*N*%	*N*%	*N*%	**0.8756**
Mean (SD)	60 (13)	59 (11)	60 (15)	
Median [Min; Max]	59 [37; 92]	58 [40; 86]	60 [34;93]	
Unknown	0	0	0	
Histology				**0.759**
Ductal	39 79.6	42 85.7	41 89.1	
Lobular	7 14.3	5 10.2	4 8.7	
Other*	3 6.1	2 4.1	1 2.2	
Unknown	0 0	0 0	0 0	
Margin status				**0.669**
Negative	46 94	46 93.9	43 93.5	
Positive	1 2	2 4.1	0 0	
Close < 1 mm	1 2	1 2	1 2.2	
Not applicable	1 2	0 0	2 4.3	
pT stage				**0.996**
T0-T1	42 85.71	41 83.67	38 82.6	
T2	6 12.24	7 14.29	7 15.2	
T3	0 0	0 0	0 0	
T4	1 2.04	1 2.04	1 2.2	
Missing	0 0	0 0	0 0	
pT stage				**0.999**
T0-T2	48 97.96	48 97.96	45 97.8	
T3-T4	1 2.04	1 2.04	1 2.2	
Missing	0 0	0 0	0 0	
pN stage				**0.981**
0	38 77.55	38 77.55	35 76.1	
1	11 22.45	11 22.45	11 23.9	
Unknown	0 0	0	0 0	
Tumor SBR grade				**0.867**
1	9 18.37	11 22.45	3 6.5	
2	28 57.14	26 53.06	12 26.1	
3	8 16.33	8 16.33	2 4.3	
Not applicable	4 8.16	4 8.16	0 0	
Unknown	0 0	0 0	29 63	
Estrogen receptor status				**0.913**
Positive	39 79.59	39 79.59	38 82.6	
Negative	10 20.41	10 20.41	8 17.4	
Unknown	0 0	0 0	0 0	
HER2 status				**0.552**
Positive	9 18.37	8 16.33	9 19.6	
Negative	37 75.51	38 77.55	37 80.4	
Not applicable	3 6.12	3 6.12	0 0	
Unknown	0 0	0 0	0 0	

*Micropapillary, medullary, metaplasic, mucinous,or tubular component

*IBC* invasive breast cancer, *SD* standard deviation, *min* minimum, *max* maximum, *SBR* Scarff-Bloom-Richardson

A p-value < 0.05 was considered statistically significant

### Comparison of locoregional and systemic therapies received by the three groups for their IBC

The treatments received by three groups of patients for their IBC are summarized in Table 3. LIR in CIS-LIR and IBC-LIR groups were mainly treated by mastectomy (in, respectively, 63.3 and 65.2%), whereas de novo IBC were mostly treated by lumpectomy (69.4%, *p* < 0.0001). There was no difference between the rate of chemotherapy (neoadjuvant or adjuvant) or adjuvant endocrine therapy received by the three groups. Logically, patients from de novo IBC group were significantly more frequently treated by radiotherapy than the other groups (*p* < 0.0001).

**Table 3:** Therapies received by the 3 groups of patients for their IBC

	CIS-LIR group (*N* = 49)	de novo IBC group (*N* = 49)	IBC-LIR group (*N* = 49)	*p*-value
	*N*%	*N*%	*N*%	
Neoadjuvant chemotherapy (NA)				**0.607**
Yes	3 6.1	3 6.1	5 10.9	
No	46 93.9	46 93.9	41 89.1	
Surgery				** < 0.0001**
Lumpectomy	17 34.7	34 69.4	3 6.5	
Mastectomy	31 63.3	15 30.6	30 65.2	
Parietal surgery	0 0	0 0	7 15.2	
Axillary lymph node dissection	0 0	0 0	4 8.7	
Not applicable	1 2	0 0	2 4.3	
Adjuvant chemotherapy				**0.611**
Yes	18 36.7	14 28.6	17 37	
No	31 63.3	35 71.4	29 63	
Chemotherapy (NA or adjuvant)				**0.422**
Yes	20 40.8	16 32.7	21 45.7	
No	29 59.2	33 67.3	25 54.3	
Adjuvant radiotherapy				** < 0.0001**
Yes	20 40.8	42 85.7	14 30.4	
No	29 59.2	7 14.3	32 69.6	
Adjuvant endocrine therapy				**0.776**
Yes	34 69.4	33 67.3	34 73.9	
No	15 30.6	16 32.7	12 26.1	

A p-value < 0.05 was considered statistically significant

### Comparison of distant-relapse-free survival (D-RFS) and overall survival (OS) between the three groups

Median follow-up from the date of diagnosis of the IBC (de novo IBC, and the second tumor for CIS-LIR and IBC-LIR groups) was 8.3 years (95% CI [6.42; 9.89] years) overall. In each group, the median follow-up from the diagnosis of IBC was 8.30 years (95% CI [6.41; 9.89] years) in CIS-LIR group; 8.34 years (95% CI [7.66; 11.63] years) in de novo IBC group; and 8.28 years from the diagnosis of the second invasive tumor (95% CI  [5.12; 10.48] years) for IBC-LIR group.

The median time between the first tumor (CIS in CIS-LIR group, or IBC in IBC-LIR) and LIR was 6.97 [1.01, 16.09] years in CIS-LIR group, and 5.34 [1.42, 15.22] years in IBC-LIR group; there was no difference between groups (*p* = 0.3194).

Among the 144 patients in the overall matched population, 8 (16.33%), 11 (22.4%), and 12 women (26.09%), respectively, in CIS-LIR, de novo IBC, and IBC-LIR groups had the primary endpoint (distant metastasis or death) (*p* = 0.503).

**Fig. 1:**
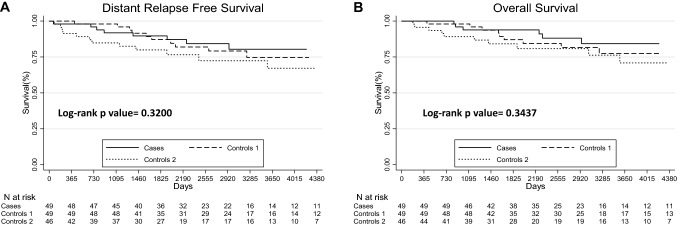
Kaplan–Meier survival curves for distant relapse-free survival and overall survival for the 3 groups of patients. **A** Distant relapse-free survival, **B** overall survival

There was no difference in D-RFS (Fig. 1A) between the 3 groups (*p* = 0.3200). Five-year and 10-year D-RFS were, respectively, 90% (95% CI [0.77; 0.96]) and 80% (95% CI [0.63; 0.90]) for CIS-LIR group, 87% (95% CI [0.73; 0.94]) and 74% (95% CI [0.57; 0.86]) for de novo IBC group, and 80% (95% CI [0.65; 0.90]) and 67% (95% CI  [0.47; 0.80]) for IBC-LIR group.

Among the 144 patients, 6 (12%) patients in CIS-LIR group, 9 (18%) in de novo IBC group and 10 (21.74%) in IBC-LIR died (*p* = 0.462). There was no difference in OS (Fig. 1B) between the 3 groups (p = 0.3437). Five-year and 10-year OS were, respectively, 94% (95% CI [0.82; 0.98]) and 84% (95% CI [0.67; 0.93]) for CIS-LIR group; 87% (95% CI  [0.73; 0.94]) and 77% (95% CI  [0.60; 0.88]) for de novo IBC group, and 84% (95% CI [0.69; 0.92]) and 71% (95% CI  [0.50; 0.84]) for IBC-LIR group.

### Comparison of D-RFI between the three groups

Among the 144 patients comprising the overall matched population, 6 (12.24%), 3 (6.12%), and 6 (13.04%) patients presented distant metastasis, respectively, in CIS-LIR, de novo IBC, and IBC-LIR groups (*p* = 0.476). There was no statistically significant difference in D-RFI between the 3 groups (*p* = 0.2357).

## Discussion

To the best of our knowledge, this is the first study to compare the outcomes between patients who experience IBC after prior ipsilateral CIS, patients with de novo invasive breast cancer, and patients with LIR of breast carcinoma after a primary ipsilateral IBC. The originality of our study is that it uses data from an exhaustive population-based registry data, with strict matching on patient data, as well as on clinical and pathological tumor characteristics, including tumor size, node stage, tumor grade, ER, and HER2 status.

Concerning the prognosis of LIR following CIS treatment, existing data are conflicting and depend on the type of surgery and the proportion of patients receiving adjuvant systemic therapy at the time of invasive relapse. In the literature, ipsilateral LIR after CIS occurs in 7.2 to 15.1% of cases in randomized trials [15].

In our series, at the time of relapse, most patients were menopaused, and mean age was 60 years. The IBCs mainly harbored favorable clinicopathologic characteristics, with the majority of cases being the ductal subtype, stage I (pT1 N0), SBR grade I-II, ER positive, and HER2 negative. These data are in accordance with the previous studies by Romero [8], Silverstein [7], and Sopik [9], where patients with ILR after prior ipsilateral DCIS mainly had same good prognosis features (stage I tumors, SBR grade II, and positive ER status).

In our study, D-RFS and OS at 5 years were, respectively, 90 and 94% in patients who experienced IBC after initial CIS, 80 and 84%, respectively, at 10 years. Overall survival rates of this magnitude could be interpreted as poor, but are in fact comparable with other studies in the literature: 82% at 10 years reported by Romero et al. [8], 82.1% at 8 years by Silverstein [7], and 92% at 8 years by Solin [16]. In these previous series, similar results were observed for D-RFS: 80% at 10 years for Romero [8], 72.9% at 8 years for Silverstein [7], 85% at 12 years for Lee [2], and 89% at 8 years for Solin [16]. Moreover, our analyses concerning D-RFI show that many deaths in fact occur without a distant relapse event (mortality not related to breast cancer). Unfortunately, the data collected in our population-based registry do not enable us to identify the cause of death, therefore precluding calculation of the BCSS.

Several studies published before ours were in favor of worse prognosis for IBC when following ipsilateral CIS [2, 8, 17–19]. We show here that when patients are matched on main clinical and pathologic characteristics, the long-term prognosis of LIR after CIS is not significantly different from de novo IBC, or even from ipsilateral LIR following a first IBC. However, ipsilateral LIR following a first IBC carries particularly poor prognosis. Concerning these patients in our study (IBC-LIR group), D-RFS at 5 years was better than rates reported in the literature: 80% for our study, compared to 52.9% for Yu et al. [20], 45% for Montagna et al. [21], and 51% for Wapnir et al. [22]. OS at 5 years, at 84%, was also better for our IBC-LIR patients, compared to 68% reported by Yu [20], 71% by Montagna [21], and 60% by Wapnir and al [22]. Importantly, it should be noted that these apparently better outcomes can be explained by favorable clinicopathologic characteristics used for matching with CIS-LIR patients.

Another explanation of the better outcomes observed in our series for all three groups could be the higher rate of adjuvant systemic therapy, despite apparently favorable clinicopathologic tumor features. For example, the rate of adjuvant chemotherapy in our study for the treatment of ipsilateral IBC recurrence is higher than in most other studies: 45.7% for IBC-LIR group of our study versus 26.9% in Montagna’s cohort [21]. We observed a similar tendency for adjuvant endocrine therapy, where the rate was 73.9% in our IBC-LIR, higher than the 58.8% reported by Yu [20] and 51.2% by Montagna [21]. The benefit of adjuvant endocrine therapy after locoregional recurrence (LRR) has been well established. The trial conducted by Waeber et al. [23] compared tamoxifen with observation in 167 patients who underwent radical surgery and radiotherapy for post-mastectomy LRR. After a median follow-up of 11 years, tamoxifen therapy significantly improved progression-free survival. However, this beneficial effect did not translate into a detectable OS advantage. On the other hand, the utility of adjuvant chemotherapy for women who experience a LRR still remains an open question. The CALOR trial [24] reported that the administration of adjuvant chemotherapy after isolated IBC locoregional recurrence significantly improved DFS, but only for patients with ER-negative tumors.

This high rate of adjuvant systemic treatments was also observed in CIS-LIR group of our study (despite apparently favorable clinicopathologic characteristics), and there was no statistically significant difference between the 3 groups in terms of administration of adjuvant chemotherapy or endocrine therapy. This could also explain the low rate of metastatic relapses observed in our study, regardless of the group.

In order to improve the prognosis of patients who are at higher risk of relapse, several new treatments are progressively being incorporated into the adjuvant armamentarium. In patients with ER-positive tumors, like the vast majority of our patients, CDK-4/6 inhibitors have improved survival in the metastatic setting, and are now being tested in the adjuvant setting, with encouraging preliminary results for patients at highest risk of relapse [25]. In patients with isolated resected invasive locoregional recurrence of ER-positive/HER2-negative breast cancer, the POLAR trial is currently being conducted to determine the effectiveness of the addition of palbociclib (a CDK-4/6 inhibitor) to adjuvant endocrine therapy *versus* endocrine therapy alone.

It is noteworthy that in our study, although excluded from our analyses, we observed a high rate (10.9%) of disease discovered at a metastatic stage at the time of LIR post-CIS. This rate of metastatic disease discovered contemporaneously with IBC was highest among patients with LIR following CIS, compared to the other clinical situations.

Time to LIR is the most frequently reported prognostic factor for DFS and OS in patients with breast cancer with LIR. Numerous studies have reported that poor DFS and OS are associated with a short time interval between the initial diagnosis and LIR, namely less than 2 years for Yu and al [20], or within five years for Witteven and al [19]. Therefore, early local recurrence has to be considered as a warning sign of more aggressive disease. The median time interval in IBC-LIR group of our study was 5.34 years, compared to 3.4 years in the cohort reported by Yu [20], and 2.6 years in Montagna’s cohort [21]. This may partially explain the differences in observed outcomes between studies. Importantly, there was no difference in the time interval between the initial disease and LIR in CIS-LIR and IBC-LIR groups of our study, suggesting the absence of bias due to different aggressive entities.

Our study had some limitations. First, it was a retrospective analysis, and secondly, there was a relatively small number of patients included in each group, and the resulting lack of power for statistical analyses. Conversely, the strength of our study lies in the triple matching of our cohort at relapse, rendering our groups comparable, and the fact that the data come from a population-based registry and are therefore representative of an entire geographical area.

In conclusion, ILR after CIS is usually considered a concerning event, since it represents a potentially life-threatening progression, with cancer-specific mortality rates close to those of stage IIA breast cancer in some studies [2, 7]. However, in our study, prior CIS does not appear to impact per se long-term outcome of IBC, compared to other IBC clinical presentations (namely de novo IBC, or LIR after a first IBC), when strict matching for clinical and pathologic characteristics is applied, and at least when comparable adjuvant systemic treatments (endocrine therapy and chemotherapy) are prescribed.

However, ipsilateral LIR following CIS must be given due to consideration by clinicians, even in case of apparently favorable clinical and pathologic tumor features, especially because of the high proportion of patients with contemporary metastases at the diagnosis of LIR, as observed in our study. Due to the heterogeneity in initial treatment and at relapse, further studies with larger patient populations and better biological characterization of these different entities are warranted to better understand the specificities and the prognosis of each of these breast cancer subtypes.

## Data Availability

All the data analyzed in this study are included in this article or in the associated supplementary files.

## Abbreviations

AJCC: American Joint Committee on Cancer
ANOVA: Analysis of variance
ASCO: American Society of Clinical Oncology
BCSS: Breast cancer-specific survival
CALOR trial: Chemotherapy for isolated locoregional recurrence of breast cancer trial
CAP: College of American Pathologists
CDK-4/6: Cyclin-dependent kinase 4/6
CEP: Chromosome enumeration probe
CI: Confidence interval
CIS: Carcinoma in situ
CNIL: Commission nationale de l’informatique et des libertés
DCIS: Ductal carcinoma in situ
DFI: Disease-free interval
D-RFI: Distant relapse-free interval
D-RFS: Distant relapse-free survival
ER: Endocrine receptor
ET: Endocrine therapy
FISH: Fluorescence in situ hybridization
HER2: Human epidermal growth factor receptor-2
HR: Hormone receptor
IHC: Immunohistochemistry
IBC: Invasive breast cancer
IL(B)R: Invasive local (breast) recurrence
LCIS: Lobular carcinoma in situ
LIN1-2: Lobular intraepithelial neoplasia 1–2
LIR: Local invasive recurrence
LRR: Locoregional recurrence
NA: Neoadjuvant chemotherapy
OS: Overall survival
PFS: Progression-free survival
POLAR trial: Palbociclib for HR-positive/HER2-negative isolated locoregional recurrence of breast cancer.
SBR: Scarff-Bloom-Richardson
SD: Standard deviation
TNM: Tumor, node, and metastasis

## References

[1] Cutuli B, Lemanski C, De Lafontan B, Chauvet M-P, De Lara CT, Mege A (2020). Ductal carcinoma in situ: a french national survey. analysis of 2125 patients. Clin Breast Cancer.

[2] Lee LA, Silverstein MJ, Chung CT, Macdonald H, Sanghavi P, Epstein M (2006). Breast cancer–specific mortality after invasive local recurrence in patients with ductal carcinoma-in-situ of the breast. Am J Surg.

[3] Donker M, Litière S, Werutsky G, Julien J-P, Fentiman IS, Agresti R (2013). Breast-conserving treatment with or without radiotherapy in ductal carcinoma in situ: 15-year recurrence rates and outcome after a recurrence, from the EORTC 10853 randomized phase iii trial. J Clin Oncol.

[4] Lagios MD (1990). Duct carcinoma in situ: pathology and treatment. Surg Clin North Am.

[5] Silverstein MJ, Barth A, Poller DN, Gierson ED, Colburn WJ, Waisman JR (1995). Ten-year results comparing mastectomy to excision and radiation therapy for ductal carcinoma in situ of the breast. Eur J Cancer.

[6] Cutuli B, Cohen-Solal-le Nir C, de Lafontan B, Mignotte H, Fichet V, Fay R (2002). Breast-conserving therapy for ductal carcinoma in situ of the breast: the French cancer centers’ experience. Int J Radiat Oncol Biol Phys.

[7] Silverstein MJ, Lagios MD, Martino S, Lewinsky BS, Craig PH, Beron PJ (1998). Outcome after invasive local recurrence in patients with ductal carcinoma in situ of the breast. J Clin Oncol.

[8] Romero L, Klein L, Ye W, Holmes D, Soni R, Silberman H (2004). Outcome after invasive recurrence in patients with ductal carcinoma in situ of the breast. Am J Surg.

[9] Sopik V, Iqbal J, Sun P, Narod SA (2016). Impact of a prior diagnosis of DCIS on survival from invasive breast cancer. Breast Cancer Res Treat.

[10] Giuliano AE, Connolly JL, Edge SB, Mittendorf EA, Rugo HS, Solin LJ, Weaver DL, Winchester DJ, Hortobagyi GN (2017). Breast cancer—major changes in the American Joint Committee on Cancer eighth edition cancer staging manual. CA Cancer J Clin.

[11] Elston CW, Ellis IO.(2002) Pathological prognostic factors in breast cancer. I. The value of histological grade in breast cancer: experience from a large study with long-term follow-up. C. W. Elston & I. O. Ellis. Histopathology 1991; 19; 403–410. Histopathology. (3A):151–2, discussion 152–153. 12405945

[12] Wolff AC, Hammond MEH, Hicks DG, Dowsett M, McShane LM, Allison KH (2013). Recommendations for human epidermal growth factor receptor 2 testing in breast cancer: american society of clinical oncology/college of american pathologists clinical practice guideline update. J Clin Oncol.

[13] Gourgou-Bourgade S, Cameron D, Poortmans P, Asselain B, Azria D, Cardoso F (2015). Guidelines for time-to-event end point definitions in breast cancer trials: results of the DATECAN initiative (Definition for the Assessment of Time-to-event Endpoints in CANcer trials). Ann Oncol.

[14] Shuster JJ (1991). Median follow-up in clinical trials. J Clin Oncol Off J Am Soc Clin Oncol.

[15] Prise en charge du carcinome canalaire in situ /Questions d’actualité. Recommandations et référentiels, INCa, septembre 2015^©^. Available from: https://www.e-cancer.fr/Expertises-et-publications/Catalogue-des-publications/Prise-en-charge-du-carcinome-canalaire-in-situ-Questions-d-actualite-Rapport-integral

[16] Solin LJ, Fourquet A, Vicini FA, Haffty B, Taylor M, McCormick B (2001). Salvage treatment for local recurrence after breast-conserving surgery and radiation as initial treatment for mammographically detected ductal carcinoma in situ of the breast. Cancer.

[17] Smith TE, Lee D, Turner BC, Carter D, Haffty BG (2000). True recurrence vs new primary ipsilateral breast tumor relapse: an analysis of clinical and pathologic differences and their implications in natural history, prognoses, and therapeutic management. Int J Radiat Oncol.

[18] Haffty BG, Carter D, Flynn SD, Fischer DB, Brash DE, Simons J (1993). Local recurrence versus new primary: clinical analysis of 82 breast relapses and potential applications for genetic fingerprinting. Int J Radiat Oncol.

[19] Witteveen A, Kwast ABG, Sonke GS, IJzerman MJ, Siesling S. (2015) Survival after locoregional recurrence or second primary breast cancer: impact of the disease-free interval. PLoS ONE [Internet]. [cited 2020 Jul 4];10(4). Available from: https://www.ncbi.nlm.nih.gov/pmc/articles/PMC4393268/10.1371/journal.pone.0120832 PMC4393268 25861031

[20] Yu C-C, Kuo W-L, Shen S-C, Chou H-H, Lo Y-F, Yu M-C (2020). Prognostic study for isolated local recurrence operated with salvage excision in hormone-receptor-positive patients with invasive breast cancer after primary breast surgery. Biomed.

[21] Montagna E, Bagnardi V, Rotmensz N, Viale G, Renne G, Cancello G (2012). Breast cancer subtypes and outcome after local and regional relapse. Ann Oncol.

[22] Wapnir IL, Anderson SJ, Mamounas EP, Geyer CE, Jeong J-H, Tan-Chiu E (2006). Prognosis after ipsilateral breast tumor recurrence and locoregional recurrences in five national surgical adjuvant breast and bowel project node-positive adjuvant breast cancer trials. J Clin Oncol.

[23] Waeber M, Castiglione-Gertsch M, Dietrich D, Thürlimann B, Goldhirsch A, Brunner KW (2003). Adjuvant therapy after excision and radiation of isolated postmastectomy locoregional breast cancer recurrence: definitive results of a phase III randomized trial (SAKK 23/82) comparing tamoxifen with observation. Ann Oncol.

[24] Wapnir IL, Price KN, Anderson SJ, Robidoux A, Martín M, Nortier JWR (2018). Efficacy of chemotherapy for ER-negative and ER-positive isolated locoregional recurrence of breast cancer: final analysis of the CALOR trial. J Clin Oncol.

[25] Harbeck N, Rastogi P, Martin M, Tolaney SM, Shao ZM, Fasching PA (2021). Adjuvant abemaciclib combined with endocrine therapy for high-risk early breast cancer: updated efficacy and Ki-67 analysis from the monarchE study. Ann Oncol.

